# Research on China Cities’ Total Factor Productivity of Carbon Emission: Based on Decoupling Effect

**DOI:** 10.3390/ijerph19042007

**Published:** 2022-02-11

**Authors:** Fang Chen, Tao Zhao, Di Wang

**Affiliations:** 1 College of Management and Economics, Tianjin University, Tianjin 300072, China; chfanghr@163.com (F.C.); mengh905@163.com (T.Z.); 2 School of Accountancy, Beijing Wuzi University, Beijing 101149, China

**Keywords:** China’s cities, carbon emission, total factor productivity, decoupling effect

## Abstract

Economic development depends on energy consumption, which is a major source of carbon emission. How to achieve economic decarbonization has become one of the key questions urgently needing to be solved on the road of carbon peak and carbon neutral development in China. Advancing total factor productivity (TFP) of carbon emission is an important way to promote economic decarbonization. For the carbon emission TFP, current research is mainly conducted from province level or an industry perspective, and studies its deference with various geographical locations, economic development levels, urbanization levels, etc., lacking the research that combines the decoupling effect to carbon emission TFP. The carbon emission TFP of Chinese cities and how to improve it remain unclear. Therefore, based on Tapio decoupling theory, this paper firstly analyzed the decoupling effect of China’s 284 cities from 2005 to 2019, and aggregated the cities into four groups according to the decoupling effect. Then, using the DEA–Malmquist index, this paper researched the carbon emission TFP and its driving factors based on the aggregation. The result shows that weak decoupling is the main decoupling status in China. As a whole, carbon emission TFP of Chinese cities does not perform well, but it shows a growth trend over time. Strong decoupling cities outperform expansive negative decoupling cities on carbon emission TFP. Technical change and pure technical efficiency change have inhibiting effect and promoting effect on carbon emission TFP, respectively, which are the main factors for the difference of carbon emission TFP between strong decoupling cities and expansive negative decoupling cities. Based on these findings, some common but differentiated recommendations are provided for improving Chinese cities’ carbon emission TFP.

## 1. Introduction

In the past century, human activities have caused the global average temperature to rise by 1 °C [1], posing a serious threat to the ecological environment, economic development, and human health. Globally, there is an urgent need to reduce carbon emissions. As the world’s second economy and the most populous country, the Chinese government actively responds to the call for environmental protection and emission reduction and has made a large effort. In 2015, the Chinese government stated in the Paris Climate Agreement that, by 2030, China’s carbon intensity will be reduced by 60–65% compared with 2005 levels. In the outline of the 14th Five-Year Plan in 2021, the Chinese government once again emphasized the achievement of emission peaking goal by 2030 and the carbon neutrality by 2060.

Economic development depends on energy consumption, which is a major source of carbon emission [2,3]. How to decarbonize the economy by improving efficiency and productivity is an important issue regarding the achievement of emission peaking and carbon neutrality.

Total factor productivity (TFP) was used to show the contribution of technological progress to economic growth other than the input of various factors [4]. However, the traditional measures of TFP did not account for environmental pollution, especially carbon emissions. Considering carbon emissions, the TFP correspondingly become carbon emission TFP (also named green productivity), which reflects the dynamic changes of carbon emission efficiency, and the contribution of technological progress to the dynamic changes. Researching TFP of carbon emission and its driving factors will lay a foundation for improving carbon emission efficiency, which in turn promotes economic decarbonization.

In addition, the decoupling effect of economic development and carbon emissions is an important index to measure the degree of economic decarbonization. With disparate degree of economic decarbonization, TFP of carbon emission and their influencing factors may differ. Therefore, it is of great significance to study the TFP of carbon emission combing with decoupling effect, which may provide a theoretical basis for differentiated green and low-carbon transformation.

Cities are the main body of economic development and the main place for human social and economic activities [5,6], which cause massive carbon emissions. Additionally, due to China’s vast land and resource endowments, China’s cities have experienced disparate developmental stages, facing various levels of decarbonization. Based on city levels, studying the decoupling effect can provide in-depth understanding on the economic decarbonization in Chinese cities, while studying the TFP of carbon emission can provide theoretical support for cities to advance their carbon emission efficiency and promote their green and low-carbon transformation.

Against this background, this paper aims to study China cities’ decoupling effect and TFP of carbon emission. The contribution of this research includes three aspects: firstly, the decoupling effect will be calculated to verify China cities’ decoupling statuses; secondly, China cities’ TFP of carbon emission will be analyzed; and thirdly, the multi-region comparisons of TFP drivers for China’s cities will be studied. After that, China’s 284 cities are aggregated into four groups according to the varied decoupling statuses.

The structure of the paper is conducted as follows. “Literature review” presents the literature review. “Methodologies and data sources” introduces the Tapio decoupling model, TFP model, and the data sources. “Results and discussion” presents the results and discussion about decoupling statuses, carbon emission TFP, and its drivers. “Conclusion and recommendations” concludes and provides some policy recommendations.

## 2. Literature Review

Malmquist index is often used to measure TFP. Considering environmental factors, the TFP of energy and environment is researched by many scholars.

Chiu et al. studied the production-environmental TFP of G20 countries based on Malmquist index [7], and showed that the production-environmental efficiency of these countries had decreased somewhat and still had a large space for improvement. Makridou et al. combined the DEA model and Malmquist index to study the energy environmental efficiency and TFP of five energy-intensive industries in 23 EU countries [8], and the results showed that the energy efficiency of all sectors was improved during the study period, and technical change played a driving role in the improvement of TFP of energy environment. In addition, Sueyoshi and Goto also used the Malmquist index to study the environmental TFP of oil companies worldwide [9].

In a Chinese context, the research on TFP of energy and environment have also achieved abundant and meaningful results. Wang et al. used Malmquist–Luenberger index to study the energy TFP of China’s provinces under different scenarios [10], while Sueyoshi et al.used Malmquist index to study the TFP of regional development and industrial pollution control in China from the provincial level [11]. By using Malmquist index, Tang et al.researched the TFP of environmental regulation in China from provincial level [12], the results showed that environmental regulation TFP enjoyed the agglomeration effect, and at the same time, technical progress had the greatest contribution to TFP of environmental regulation. Using the super efficiency DEA model and Malmquist index, Ma et al. studied the energy and environmental efficiency of three provinces in Northeast China [13], and conducted decomposition analysis of their total factor production efficiency. The results showed that energy and environmental efficiency were different from TFP, and Heilongjiang province had the highest TFP.

The energy environmental TFP of various sectors or industries in China had also attracted scholars’ attentions. Zhao et al. studied the environmental TFP and its dynamic change of the industry in 10 provinces [14], and then Chen et al. conducted their study from province level in China [15]. They found that the environmental TFP of most eastern provinces were higher than other provinces, and technical change contributed most to the improvement of environmental TFP. Li and Lin studied the green TFP of China’s manufacturing industry [16], and the results showed that provinces adopting sustainable energy conservation and emission reduction strategies had higher green TFP. Zhang et al. studied the TFP of carbon emission of China’s provincial transportation sector [17], while Fei and Lin studied the energy environmental TFP of China’s agricultural sector with considering regional differences [18]. They found that the overall carbon emission efficiency of China’s transportation sector decreased by 32.8% while energy environmental TFP of China’s agricultural sector was at a low level with large regional differences. Additionally, technical regression was the main reason for the non-ideal TFP.

Some scholars conducted their research on cities with a certain characteristic in China. Liu and Jie studied the green TFP of 18 cities in Sichuan province of China [19]. The industrial green efficiency in Sichuan province was low, but in recent years industrial green TFP is greater than 1; green technical efficiency showed a trend of rapid growth, but the technology progress inhibited the trend. Zhou used Malmquist index to study the TFP of carbon emission in 14 cities and states in Hunan Province [20]. They found the overall level of carbon emission efficiency in Hunan Province was low, but it enjoyed an upward trend, and the scale effect and technology effect increased the difference among cities and states.

In conclusion, the research on China’s TFP of environment or carbon emissions and its driving factors were mainly conducted from province level or industry perspective. There is a lack of quantitative research on China’s TFP of carbon emission by city level. However, cities are the main body of economic development and the main place generating carbon emissions; advancing the TFP of carbon emission of Chinese cities can promote the economic decarbonization in China. Thus, this paper will research the TFP of carbon emission on the city level in China. In addition, when studying the driving factors of carbon emission TFP, the current research studied its deference with different geographical locations, economic development levels, urbanization levels, etc., which are lacking in the research that combines the decoupling effect to carbon emission TFP. Therefore, based on the decoupling effect of economic development from carbon emissions, this paper will firstly classify the Chinese cities into four kinds, and then study the carbon TFP and its driving factors for each kind of city.

## 3. Methodologies and Data Sources

### 3.1. City Level Emission Accounting Method

Following the “Intergovernmental Panel on Climate Change (IPCC) Carbon Emission Calculation Guidelines 2007” [21], this paper calculated the total energy-related carbon emissions of 284 prefecture level cities and municipal districts in China from 2005 to 2019. Referencing the research of Liu et al. [22], Xie et al. [23], and Zhao et al. [24], this paper summarized the sources of emissions in Chinese cities into five aspects: artificial coal gas, natural gas, liquefied petroleum gas (LPG), central heating, and electricity. The total emissions here included direct emissions from fossil energy consumption and indirect emissions from heat and electricity consumption [25]. The carbon emissions are calculated as follows:

(1) $$CO_{2}^{t}=CO_{2,F}^{t}+CO_{2,H}^{t}+CO_{2,E}^{t}$$

(2) $$CO_{2,F}^{t}=\underset{i}{\sum }CO_{2i}^{t}=\underset{i}{\sum }E_{i}^{t}\times EF_{i}\times O_{i}$$

(3) $$CO_{2,H}^{t}=HEC^{t}\times AHF^{t}$$

(4) $$CO_{2,E}^{t}=HEC^{t}\times AHF^{t}$$

Among them, $CO_{2}^{t}$ is the total carbon emission in year *t* (10^4^ *ton*). $CO_{2,F}^{t}$ refers to the direct carbon emission from fossil energy consumption in year *t* (10^4^ *ton*). $CO_{2,H}^{t}$ is the indirect carbon emission from heating power consumption in year *t* (10^4^ *ton*). $CO_{2,E}^{t}$ represents the indirect carbon emission from electricity consumption in year *t* (10^4^ *ton*). The Equation (2) is used to calculate the direct carbon emission from fossil energy consumption, where $CO_{2i}^{t}$ represents the carbon emission from the *i*-th fossil energy consumption (10^4^ *ton*). $E_{i}^{t}$ is the consumption of fossil energy *i (TJ)*. $EF_{i}$ represents the IPCC recommended carbon emission factor of fossil energy *i* (*ton*/*TJ*), and $O_{i}$ refers to the oxidation rate of fossil energy *i*.

Equations (3) and (4) are used to calculate the indirect carbon dioxide produced by heat consumption and electricity consumption, where $HEC^{t}$ and $ELC^{t}$ are, respectively, the heat consumption and electricity consumption in year *t*, in units of 10^10^ *KJ* and 10^8^ *KWh*. $AHF^{t}\;$ and $AEF^{t}\;$ are the average carbon emission coefficient of heat and electricity in year *t*, respectively, in 10^4^ *ton/*10^10^ *KJ* and 10^4^ *ton/*10^8^ *KWh*. This paper assumes that the average carbon emission coefficient of heat of a city is equal to the average carbon emission coefficient of heat of the province the city belongs to. Additionally, a similar assumption is made for the average carbon emission coefficient of electricity. Referring to Xu et al. [25], the average carbon emission coefficient of heat and electricity of each province can be evaluated as follows:

(5) $$AHF^{t}=\frac{\sum_{i}CO_{2i,h}^{t}}{HG_{fossil}^{t}}$$

(6) $$AEF^{t}=\frac{\sum_{i}CO_{2i,e}^{t}+CO_{2h,e}^{t}}{EG_{fossil}^{t}+EG_{nuclear}^{t}+EG_{hydro}^{t}}$$

Among them, $CO_{2i,h}^{t}$ represents the carbon emissions based on fossil energy *i* in year *t* for heat generation (10^4^ *t*). $HG_{fossil}^{t}$ represents the heat generated by fossil fuel combustion in year *t* (10^10^ *KJ*). $CO_{2i,e}^{t}$ and $CO_{2h,e}^{t}$ respectively represent the carbon emissions based on fossil energy *i* and heat consumption for electricity generation in year *t* (10^4^ *t*). $EG_{fossil}^{t}$, $EG_{nuclear}^{t}$, and $EG_{hydro}^{t}$, respectively, represent the heat generated by fossil fuel combustion, nuclear combustion, and hydro combustion in year *t* (10^8^ *KWh*).

### 3.2. Tapio Decoupling Theory

In 2002, OEDC proposed the concept of decoupling, and proposed a decoupling calculation method to study the asynchronous changes of environmental damage and economy [26]. Subsequently, Tapio proposed a decoupling method based on elasticity coefficient [27], namely the Tapio decoupling theory. Due to its insensitivity to base period selection and the ability to identify different degrees of decoupling, Tapio decoupling theory and methods have been widely used in research to verify the decoupling relationship between emissions and economic development.

Following Tapio [27], the decoupling effect (DE) of economic development from carbon emissions is utilized in this research, and its 8 decoupling statuses are shown in Table 1. According to Tapio [27], the decoupling effect (DE) can be calculated as follows:

(7) DE=%C%GDP=(Ct−C0)C0(GDPt−GDP0)GDP0=ΔCC0ΔGDPGDP0

Among them, DE represents the decoupling effect of economic development from carbon emissions, %C and %GDP represent the change rates of emissions and economic development, respectively; $C^{t}$ and $C^{0}$ are emissions in period *t* and the base period (10^4^ *ton*), respectively; and $GDP^{t}$. and $GDP^{0}$ are the gross domestic product in period *t* and the base period (100 *M yuan*), respectively. ΔC and ΔGDP represent the change in emissions and *GDP* from the base period to period *t*, respectively.

### 3.3. DEA–Malmquist Index

The combination of DEA approach with the Malmquist index (DEA–Malmquist index) was firstly proposed by Färe et al. to reflect the TFP and efficiency change [28]. However, the DEA–Malmquist index in their research did not take into account the undesirable outputs. Considering environmental pollutions, Chung et al. applied the directional distance function (DDF) to show the environmental TFP [29] Then, the DDF was widely used in researching TFP and efficiency change [17,30].

According to Zhang et al. [17], for a production process considering undesirable outputs, each city has three vectors: inputs ($x\in R^{m}$), desirable outputs ($y^{g}\in R^{e}$), and undesirable outputs ($y^{b}\in R^{f}$). m,e,f are the numbers of inputs, desirable outputs, and undesirable outputs, respectively. The production technology can be described as

(8) $$P=\left\{\left(x,y^{g},y^{b}\right):x\;can\;produce\;\left(y^{g},y^{b}\right)\right\}$$

In production economic theory, finite inputs can only generate finite output. Additionally, as pollutant emissions are by-products of energy consumption, in order to make the model with both desirable and undesirable outputs reasonable, additional assumptions need to be imposed such as weak disposability. The weak disposability assumption implies that the reduction in undesirable outputs is not free, along with a proportional abatement of desirable outputs.

According to Zhang et al. [17], the directional distance function at time *t* is defined as follows:

(9) $$D^{t}\left(x^{t},{y^{g}}^{t},{y^{b}}^{t}\right)=\sup\left\{\xi :\left({y^{g}}^{t},\;{y^{b}}^{t}\right)+\xi d\in P^{t}\right\}$$

Additionally, Zhang et al. [17] defined an input–output vector $\left(x^{t},{y^{g}}^{t},{y^{b}}^{t}\right)$ at time *t* based on the t+1 technical condition as follows:

(10) $$D^{t+1}\left(x^{t},{y^{g}}^{t},{y^{b}}^{t}\right)=\sup\left\{\xi :\left({y^{g}}^{t},\;{y^{b}}^{t}\right)+\xi d\in P^{t+1}\right\}$$

where d is a direction vector. Additionally, the corresponding Malmquist index is defined as follows:

(11) $$M^{t+1}\left(x^{t+1},{y^{g}}^{t+1},{y^{b}}^{t+1},x^{t},{y^{g}}^{t},{y^{b}}^{t}\right)=\sqrt{\frac{D^{t}\left(x^{t+1},{y^{g}}^{t+1},{y^{b}}^{t+1}\right)}{D^{t}\left(x^{t},{y^{g}}^{t},{y^{b}}^{t}\right)}\times \frac{D^{t+1}\left(x^{t+1},{y^{g}}^{t+1},{y^{b}}^{t+1}\right)}{D^{t+1}\left(x^{t},{y^{g}}^{t},{y^{b}}^{t}\right)}}$$

where $D^{t}\left(x^{t},{y^{g}}^{t},{y^{b}}^{t}\right)$ and $D^{t}\left(x^{t+1},{y^{g}}^{t+1},{y^{b}}^{t+1}\right)$ are the efficiency values at time *t* based on the *t* and t+1 technical condition, respectively. $D^{t+1}\left(x^{t},{y^{g}}^{t},{y^{b}}^{t}\right)$ and $D^{t+1}\left(x^{t+1},{y^{g}}^{t+1},{y^{b}}^{t+1}\right)$ are the efficiency values at time t+1 based on the *t* and t+1 technical condition, respectively. $M^{t+1}\left(x^{t+1},{y^{g}}^{t+1},{y^{b}}^{t+1},x^{t},{y^{g}}^{t},{y^{b}}^{t}\right)\geq 1$ means TFP of carbon emission is ideal, otherwise the TFP should be improved. In this paper, Equation (11) was employed to evaluate the TFP of carbon emission.

Under the assumption of constant returns-to-scale (*CRS*), the Malmquist index can be decomposed into efficiency change (*EC*) and technical change (*TC*). According to Färe et al. (1994), under the assumption of variable returns-to-scale (*VRS*), the efficiency change (*EC*) can be decomposed into pure technical efficiency change (*PEC*) and scale efficiency change (*SEC*). The decomposed process is showing as follows:

(12) $$\begin{array}{ll} M^{t+1}\left(x^{t+1},{y^{g}}^{t+1},{y^{b}}^{t+1},x^{t},{y^{g}}^{t},{y^{b}}^{t}\right) & =\frac{D^{t+1}(x^{t+1},{y^{g}}^{t+1},{y^{b}}^{t+1}|VRS)}{D^{t}(x^{t},{y^{g}}^{t},{y^{b}}^{t}|VRS)}\\ & \times \frac{D^{t+1}(x^{t+1},{y^{g}}^{t+1},{y^{b}}^{t+1}|CRS)}{D^{t+1}(x^{t+1},{y^{g}}^{t+1},{y^{b}}^{t+1}|VRS)}\times \frac{D^{t}(x^{t},{y^{g}}^{t},{y^{b}}^{t}|VRS)}{D^{t}(x^{t},{y^{g}}^{t},{y^{b}}^{t}|CRS)}\\ & \times \sqrt{\frac{D^{t}\left(x^{t+1},{y^{g}}^{t+1},{y^{b}}^{t+1}\right)}{D^{t+1}\left(x^{t+1},{y^{g}}^{t+1},{y^{b}}^{t+1}\right)}\times \frac{D^{t}\left(x^{t},{y^{g}}^{t},{y^{b}}^{t}\right)}{D^{t+1}\left(x^{t},{y^{g}}^{t},{y^{b}}^{t}\right)}}\\ & =PEC\times SEC\times TC \end{array}$$

Among them, variables and referential meanings in Equation (12) are the same as those in Equation (11).

In this paper, the DEA–Malmquist index model, considering the unexpected output, was used to calculate the TFP of carbon emission of 284 cities in China from 2005 to 2019. According to the decomposition analysis results of MI, the TFP of carbon emission of China’s cities can be decomposed into efficiency change (*EC*) and technical change (*TC*), and the efficiency change (*EC*) can be further decomposed into pure technical efficiency change (*PEC*) and scale efficiency change (*SEC*). The calculation formulas are Equations (12) and (13). Among them, *TC* refers to the contribution to TFP caused by the introduction of advanced equipment and technology and the development of new production technology, method, theory and process flow. *PEC* refers to the contribution to TFP caused by the change of the ability and management ability of an organization to use internal resources (such as business strategy, plan and policy, etc.); and *SEC* refers to the contribution to TFP that can be caused by changes in internal scale factors (such as business capital, production capacity, etc.) and external scale factors (such as policy environment, production demand, etc.).

### 3.4. Data Source

This paper computed the TFP of carbon emission and its driving factors in China from 2005 to 2019 at the city level. Based on the division of administrative regions in China and the availability of data, the research scope of this paper includes 284 cities at and above the prefectural level in China, not including Chaohu (adjusted to county level city in 2011), Laiwu (adjusted into Jinan as Laiwu District, 2019), Lhasa, Hong Kong, Macao, and Taiwan, as well as the cities in Tibet and the cities set after 2005.

When calculating the carbon emissions of each city, all the carbon emissions are those generated by end-use energy consumption. The energy consumption data of each city comes from China Statistical Yearbook of Urban and Rural Construction and China Statistical Yearbook of Cities. The oxidation rate and recommended carbon emission factor of fossil energy are all referred to IPCC and China Statistical Yearbook of Energy.

Carbon emission data and *GDP* data are needed to calculate the decoupling effect of economic development from carbon emissions. In this paper, the *GDP* original data of China’s cities came from China Statistical Yearbook of Cities and are converted into constant prices in 2010 based on the *GDP* price index of each city.

In addition, this paper aims to study the TFP of carbon emission of 284 cities in China, so the decision units are the 284 cities in China. In the DEA–Malmquist index model, there were three input indicators for each city: labor, assets, and energy consumption. Additionally, *GDP* (Gross Domestic Product) and carbon emission are the expected output and undesirable output, respectively. The labor refers to the employed staff of each city at the end of the year. The data come from China Statistical Yearbook of Cities. The asset refers to the total input of assets in the current year, which is calculated by the perpetual inventory method. The original data come from the China Statistical Yearbook of Cities and the statistical bulletin of national economic and social development of some cities, and is converted into the constant price in 2010 based on the fixed asset price index. Energy is the end-use energy consumption of each city. The original data of energy consumption, *GDP*, and carbon emissions in DEA–Malmquist index model stay the same with above. The input and output indicators selected in the DEA–Malmquist index model is showing in Table 2.

In order to ensure the validity of the data, all data are checked with data from the Annual Database of Cities in China Economic Network Statistics Database, China City Database, China Urban–Rural Construction Database, and China Regional Economic Database. The moving average method and the average growth rate method are used in this paper when finding several missing values for a few cities.

## 4. Carbon Emission and Decoupling Effect Analysis of China’s Cities

### 4.1. Carbon Emission Analysis

**Figure 1:**
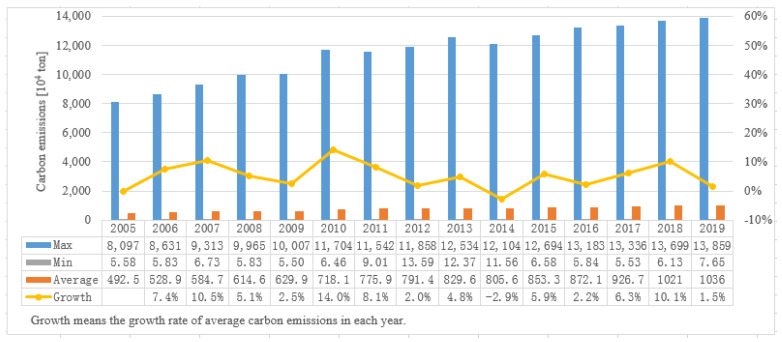
The carbon emissions of Chinese cities during 2005–2019.

Table 3 shows the cities with the largest or smallest carbon emissions in each year during the study period, and Figure 1 shows the maximum and minimum carbon emissions of Chinese cities in each years during the study period.

As shown in Table 3, Shanghai had the largest carbon emissions among all the cities during the study period, except in 2009. In 2009, the city with the largest carbon emissions was Beijing. Shanghai and Beijing, as the first-tier cities in China, take the leading position in economic scale and rank second and third respectively in population size. The huge size of the economy and population lead Shanghai and Beijing to be the biggest carbon emitters.

From 2005 to 2012, Longnan had the lowest carbon emissions, while Lijiang had the lowest carbon emissions in 2013. From 2014 to 2019, Lincang had the lowest carbon emissions. Longnan and Lincang are famous tourist attractions in Gansu province and Yunnan Province, respectively. The subtropical monsoon climate makes Longnan and Lincang enjoy a magnificent and beautiful natural scenery as well as being rich in resources, causing a unique low-carbon environment. Therefore, Longnan and Lincang have become the cities with the lowest carbon emissions in China.

As shown in Figure 1, there is a significant difference between the maximum and minimum carbon emissions among Chinese cities. The result indicates that it is of great practical significance to study carbon emissions and related issues in China from the perspective of cities.

Seeing the minimum value, the minimum carbon emissions of Chinese cities during 2005 to 2019 are basically less than 10 × 10^4^ tons. During the study period, the minimum carbon emissions of Chinese cities first increase and then decrease, indicating that low-emission cities performed well on reducing carbon emission.

At the same time, the maximum carbon emissions of Chinese cities show a gradual upward trend, reaching 13,859 × 10^4^ tons in 2019. Rapid economic development and expanding population size may be the main reasons for the rising carbon emissions in Shanghai and Beijing [31].

Compared with the maximum value, the average carbon emissions of Chinese cities are at a relatively low position. The result indicates that the carbon emissions of most Chinese cities are relatively concentrated, and there are only a few cities with carbon emissions of more than 1000 × 10^4^ tons. However, during the study period, the growth rate of average carbon emissions is basically greater than 0, showing that the carbon emissions of most cities are increasing steadily from 2005 to 2019. Therefore, the carbon reduction work in China should focus not only on high-emission cities, but also on medium-emission cities, which means improving the carbon emission efficiency of medium-emission cities and reducing their emissions.

### 4.2. Decoupling Effect Analysis

**Figure 2:**
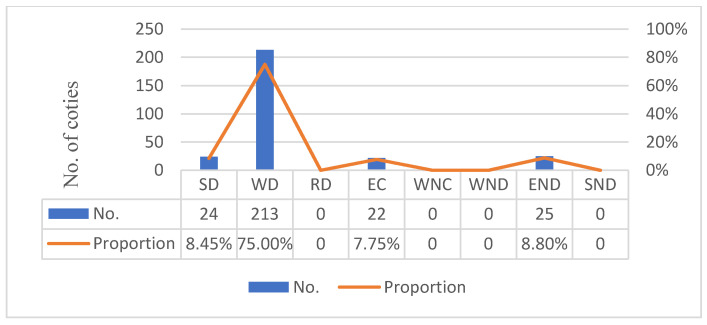
Statistics on decoupling statuses of Chinese cities from 2005 to 2019.

Adhering to the Tapio decoupling theory, this paper firstly calculated the decoupling effect of each city from 2005 to 2019, then determined the decoupling statuses of each city based on the decoupling effect value. Figure 2 shows the decoupling status analysis of China’s cities at the number of cities in the period of 2005 to 2019.

In general, among the eight logical possibilities for decoupling statuses, weak decoupling, expansive negative decoupling, strong decoupling, and expansive coupling are the ones which occur in China, with the proportion of 75%, 8.8%, 8.45%, and 7.75%, respectively.

According to the Tapio decoupling criteria for eight logical possibilities, strong decoupling means simultaneous economic growth and carbon reduction, which is the ideal decoupling. From 2005 to 2019, there are 24 cities that have achieved strong decoupling of economic development from carbon emissions. This phenomenon shows that, under certain conditions, Chinese cities have the ability to achieve the strong decoupling of economic development from carbon emissions.

Among the four relevant decoupling statuses, the number of weak decoupling cities is the largest, up to 213 and accounting for 75% to the total cities. This phenomenon shows that the weak decoupling status is the common status of Chinese cities, indicating that the weak decoupling status should be the goal of cities that have not achieved the decoupling of carbon emission from economic development.

There are 22 cities at expansive coupling, accounting for 7.75% of the total cities. For these cities, gradually achieving the decoupling economic development from carbon emissions is a key concern for the future.

There are 25 cities at expansive negative decoupling, accounting for 8.80% of the total cities, indicating that the positive economic growth of these cities during 2005 to 2019 is relying on huge energy consumption, and the reduction pressure of those cities is greater. For these cities, improving production efficiency and energy efficiency is the main concern for the future.

The cities in various decoupling statuses have great differences in social and economic characteristics; the focus on future development are disparate, too. Thus, this paper aggregated China’s 284 cities into four groups according to their decoupling status. The strong decoupling city refers to the city that enjoys strong decoupling of economic development from carbon emissions from 2005 to 2019, and so forth for weak decoupling cities, expansive coupling cities, and expansive negative decoupling cities. There are 24, 213, 22, and 25 strong decoupling cities, weak decoupling cities, expansive coupling cities, and expansive negative decoupling cities, respectively.

## 5. TFP of Carbon Emission and Its Driving Factors of China’s Cities

This section will analyze Chinese cities’ TFP of carbon emission and its driving factors based on decoupling effect, to explore the change of carbon emission efficiency. To simplify the presentation, the TFP of carbon emission in 2006 refers to the TFP from 2005 to 2006, and so forth for the other years.

### 5.1. TFP of Carbon Emission Analysis

**Figure 3:**
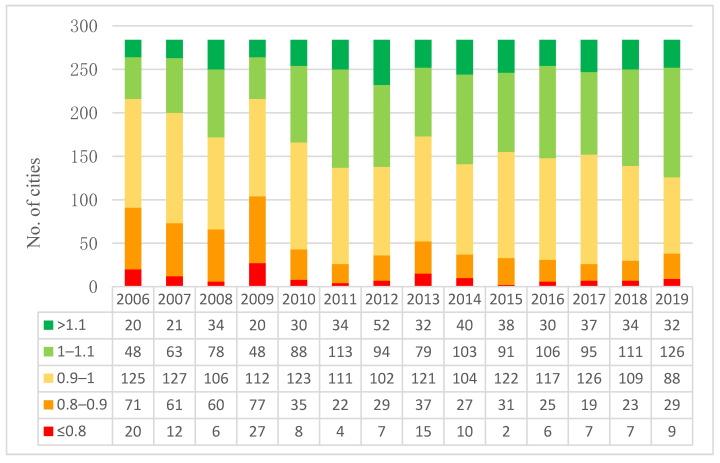
Statistics on carbon emission TFP of China’s cities during 2006–2019.

Figure 3 shows the statistical analysis of carbon emission TFP in Chinese cities during the study period. According to the quality of carbon emission TFP, the value range of carbon emission TFP is greater than zero. Additionally, the greater the TFP of carbon emission, the better. As shown in Figure 3, red means that the carbon emission TFP of the corresponding city is no more than 0.8; that is, the carbon emission efficiency is declining. Green means that the carbon emission TFP of the corresponding city is greater than 1; that is, the carbon emission efficiency shows growth.

Overall, except in 2011, 2018, and 2019, the number of cities with carbon emission TFP concentrated 0.9 to 1 is the largest, indicating that the cities with slightly decreased carbon emission efficiency are the largest over the years. In addition, the number of the cities declines first, then rises, and then declines with the progress of time, showing a trend of fluctuating decline.

As time goes on, the number of cities with carbon emission TFP greater than 1 gradually increases, indicating that, over the years, more and more cities improved their carbon emission efficiency. Among these cities, the carbon emission TFP of most cities is concentrated 1 to 1.1; that is to say, the growth of carbon emission efficiency is not large.

During 2006 to 2019, the number of cities with carbon emission TFP greater than 1.1 shows a trend of fluctuation upward. This phenomenon indicates that more and more cities in China have achieved a relatively large increase in carbon emission efficiency, and Chinese cities have the potential to achieve a relatively large increase in carbon emission efficiency.

In addition, the number of the cities with carbon emission TFP not higher than 0.8 has shown a trend of fluctuating decline, indicating that the decline on carbon efficiency in those cities has moderated.

### 5.2. The Driving Factor Analysis of the TFP

Using factor decomposition analysis method, this section studies the driving factors of carbon emission TFP of 284 cities in China, as well as the strong decoupling cities (SD cities), weak decoupling cities (WD cities), expansive coupling cities (*EC* cities), and expansive negative decoupling cities (END cities), respectively. Based on decomposition analysis method, the carbon emission TFP was decomposed into technical change index (*TC*), pure technical efficiency change index (*PEC*), and scale efficiency change index (*SEC*).

#### 5.2.1. The Driving Factors Analysis of All China’s Cities

**Figure 4:**
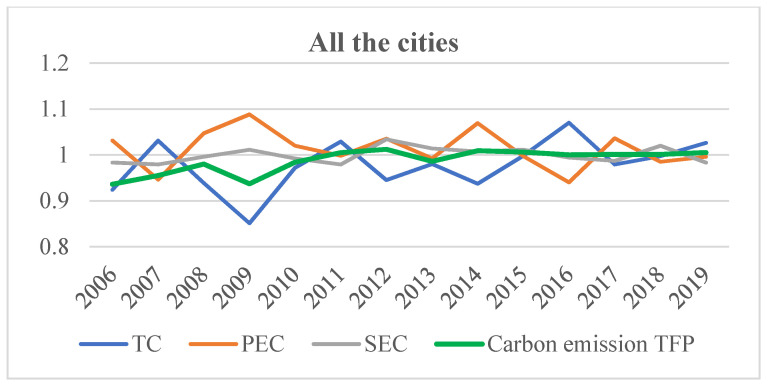
The driving factors of carbon emission TFP of China’s cities during 2006–2019.

Figure 4 shows the mean values of carbon emission TFP and its driving factors during the study period. As shown in Figure 4, the mean of carbon emission TFP shows a rise trend with fluctuation, which is consistent with the statistical analysis of carbon emission TFP in Chinese cities. Between 2006 and 2010, the mean of carbon emission TFP is less than 1, and since 2010, the mean of carbon emission TFP is mainly greater than 1 (except in 2013). This result indicates that before 2010 the carbon emission efficiency of China’s cities gradually declines as a whole, while since 2010, the carbon emission efficiency of China’s cities gradually enhances as a whole.

Technical change mainly has an inhibiting effect on carbon emission TFP in Chinese cities, and the inhibiting effect gradually decreases. As shown in Figure 4, except for 2007, 2011, 2016, and 2019, the technical change index is less than 1, indicating that, in most years, technical change inhibits the increase in carbon emission TFP. However, as time goes on, the technology change index gradually approaches 1, indicating that the inhibiting effect of technical change on carbon emission TFP of China cities gradually decreases, which is in accord with the findings obtained by Wang et al. [10] andChen et al. [15].

During 2006 to 2019, the mean value of pure technical efficiency change index fluctuates between 0.94 and 1.088. This result indicates that the promoting and inhibiting effects of pure technical efficiency change on carbon emission TFP show an alternating phenomenon over time.

As time goes on, the scale efficiency change index fluctuates between 0.979 and 1.034, indicating that the promoting and inhibiting effects of scale efficiency change on carbon emission TFP changes with time. Additionally, the effect of scale efficiency change on carbon emission TFP is not obvious.

#### 5.2.2. The Driving Factors Analysis of Cities Based on Decoupling Statuses

**Figure 5:**
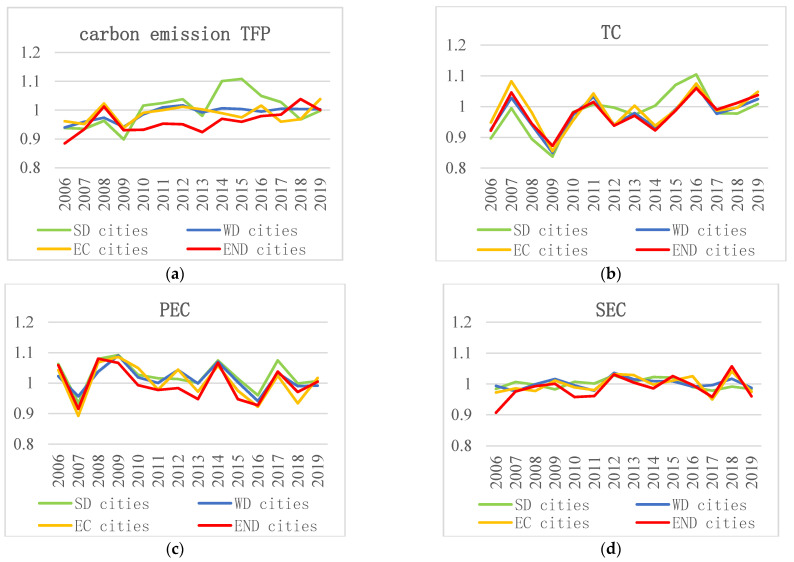
The driving factors of carbon emission TFP of each cities during 2006–2019. (**a**) the mean value of carbon emission TFP of strong decoupling (SD) cities, weak decoupling (WD) cities, expansive coupling (*EC*) cities and expansive negative decoupling (END) cities, respectively. (**b**) the mean value of technical change index (*TC*) of each kind of cities. (**c**) the mean value of pure technical efficiency index (*PEC*) of each kind of cities. (**d**) the mean value of scale technical efficiency index (*SEC*) of each kind of cities.

Figure 5 shows the mean values of carbon emission TFP and its driving factors of cities in different decoupling statuses between 2006 and 2019.

During the research period, the mean values of carbon emission TFP of cities show different trends among different decoupling statuses. First, in most years, the carbon emission TFP of strong decoupling cities is observably greater than that of expansive negative decoupling cities, showing that strong decoupling cities perform better than expansive negative decoupling cities at the growth of carbon emission efficiency, which is consistent with the description of the decoupling statuses by decoupling theory. Therefore, strong decoupling cities should try their best to maintain the continuous growth of carbon emission efficiency, while expansive negative decoupling cities should try their best to avoid the decline of carbon emission efficiency. Second, the difference of carbon emission TFP mean value between weak decoupling cities and expansive coupling cities is small, and the trend of the two cities is consistent, with a small fluctuation near base line 1. This result indicates that the average carbon emission efficiency of weak decoupling cities and expansive coupling cities does not change much. Therefore, the main task for weak decoupling cities and expansive coupling cities, at present, is to gradually promote the carbon emission efficiency by a small increase.

Between 2006 and 2019, the mean value of technical change index fluctuated greatly, which had a significant inhibiting effect on carbon emission TFP, and those effects were significantly different among cities with different decoupling statuses. As shown in Figure 5b, for all kinds of cities, the mean value of technical change index fluctuated greatly, indicating that technical change is an important factor affecting carbon emission TFP. Second, in most years, the technical change index of all kinds of cities was less than 1, showing that technical change mainly inhibits the growth of carbon emission efficiency for all kinds of cities in China. Third, there was a significant difference in the technical change index between strong decoupling cities and expansive negative decoupling cities, indicating that technical change is the most important reason for the difference in carbon emission TFP between the two kinds of cities, which is in accord with the findings obtained by Fei and Lin [18].

Pure technical efficiency change contributed significantly and positively to carbon emission TFP, and the effects are significantly diverse among cities in different decoupling statuses. As shown in Figure 5c, for all kinds of cities, the mean value of pure technical efficiency change index fluctuated greatly, indicating that the change of pure technical efficiency is an important factor affecting the carbon emission TFP, which is in accord with the findings obtained by Zhou [20]. Secondly, in most years, the mean value of pure technical efficiency index of all kinds of cities was greater than 1, indicating the positive contribution of pure technical efficiency change to carbon emission TFP. Thus, continuing the promoting effect of pure technical efficiency change is necessary to improve the carbon emission efficiency of China’s cities. Third, the mean value of pure technical efficiency change index is significantly different between strong decoupling cities and expansive negative decoupling cities, indicating that pure technical efficiency change is also an important reason for the difference on carbon emission TFP.

As shown in Figure 5d, for all kinds of cities, the fluctuation of scale efficiency change index is small, and there is no obvious difference on scale efficiency change index among cities in different decoupling statuses. The results indicate the unobvious effect on carbon emission TFP, and there is no significant difference among cities in different decoupling statuses. Continuously improving the scale efficiency is the key point for all kinds of cities in their process of improving the carbon emission TFP and promoting the growth of carbon emission efficiency.

## 6. Recommendations

Based on the abovementioned results, some recommendations are proposed to improve the carbon emission TFP in Chinese cities:

First, increase investment in research and development to improve the level of technical innovation of cities. According to Chen et al. [31], technical levels measured by patent license number has significant negative effects on carbon emission efficiency, but the effect value is small. This finding explains why technical change mainly has an inhibiting effect on carbon emission TFP in Chinese cities, and the inhibiting effect gradually decreases. Therefore, promoting the transformation of patents into productively effectively, and transforming the inhibiting effect of technical change on carbon emission TFP into promotion effect gradually, are important ways to improve the efficiency of carbon emission in Chinese cities. Therefore, on the one hand, increase the input of R&D personnel, facilities, and funds to comprehensively raise the level of technical change. On the other hand, promote the application of technical progress in social production and people’s life, emphasize the industrialization and scale of technical progress, and then improve the efficiency change by improving pure technical efficiency and scale efficiency.

Second, strengthen regional and cross-regional cooperation to achieve scale effect. Scale efficiency change has no obvious effect on carbon emission TFP, suggesting that development of most cities in China has not achieved economies of scale. Thus, promoting the scale effect of each city by regional cooperation or across regional cooperation can help China’s cities raise the level of scale efficiency change, then improve the carbon emission TFP. Therefore, on the one hand, promote regional and cross-regional technology exchanges and cooperation with help and compensation, to reduce barriers and cost of technology progress. On the other hand, promote regional and cross-regional economic interaction, to improve the scale efficiency of strong decoupling cities and weak decoupling cities as well as the technical change of expansive coupling cities and expansive negative decoupling cities.

Third, improve the capacity of organization and governance of each city. Strengthening the supervision and the guidance by environmental policies and improving the ability of cities’ comprehensive governance can help improve pure technical efficiency change, then improve the carbon emission TFP. Therefore, on the one hand, improve the capacity of cities to use internal resources by improving the governance structure, promoting the implementation and supervision of environmental policies. On the other hand, improve the comprehensive level of governance by improving the professional ability and management ability of personnel in relevant departments of the city.

## 7. Conclusions

Studying the TFP of carbon emission and its driving factors will help to improve the TFP of carbon emission in Chinese cities, then effectively achieve economic decarburization. Therefore, using the DEA–Malmquist index model, this paper analyzed the carbon emission TFP and its driving factors of Chinese cities, and discussed the deference on the driving factors after aggregating the 284 cities into strong decoupling cities, weak decoupling cities, expansive coupling cities, and expansive negative decoupling cities. The main conclusions are as follows:

First, weak decoupling is the main decoupling status in China, followed by expansive negative decoupling, strong decoupling, and then expansive coupling. Second, as a whole, the carbon emission TFP of China cities does not perform well, but it shows an increasing trend over time. Third, the value of carbon emission TFP is disparate between strong decoupling cities and expansive negative decoupling cities. Strong decoupling cities outperform expansive negative decoupling cities. At present, technical change and pure technical efficiency change have, respectively, an inhibiting effect and promoting effect on carbon emission TFP, which are the main factors for the difference of carbon emission TFP between strong decoupling cities and expansive negative decoupling cities. The contribution of scale efficiency change to carbon emission TFP is not obvious, and there is no significant difference among cities in different decoupling statuses. These results could provide a theoretical basis for differentiated green and low-carbon transformation of Chinese cities.

However, there were several limitations of this study. First, due to data limitations, only the municipal districts’ carbon emissions of 284 cities in China were considered. In China, each city is divided into counties, municipalities, and municipal districts. Therefore, the carbon emissions calculated by using the method in this paper are not the total carbon emissions of cities. The future study could take a more effective way to measure carbon emissions in China’s cities. Second, this paper studied the decoupling effect and carbon emission TFP from a macro perspective, which provides a theoretical basis for cities in China to achieve economic decarburization. However, achieving economic decarburization ultimately depends on each individual enterprise. Thus, research on enterprises’ decoupling effect and carbon emission TFP should be conducted in the future.

## Figures and Tables

**Table 1:** The Tapio decoupling criteria for eight logical possibilities.

Decoupling Statuses		∆*C*	∆*GDP*	DE	Symbol
Decoupling	Strong decoupling	<0	>0	(−∞,0)	SD
	Weak decoupling	≥0	≥0	[0,0.8]	WD
	Recessive decoupling	<0	<0	(1.2,+∞)	RD
Coupling	Expansive coupling	>0	>0	(0.8,1.2]	*EC*
	Weak negative coupling	<0	<0	(0.8,1.2]	WNC
Negative decoupling	Weak negative decoupling	<0	<0	[0,0.8]	WND
	Expansive negative decoupling	>0	>0	(1.2,+∞)	END
	Strong negative decoupling	>0	<0	(−∞,0)	SND
Source: Tapio [27] (2005)					

**Table 2:** Input and output indicators selected in the DEA–Malmquist index model.

Indicators		Definition
Inputs	Labor	The number of staff by the end of the year.
	Asset	The sum of current capital and fixed investment was calculated, then converted into 2010 constant price.
	Energy	The end-use energy consumption in the city, computed into coal equivalent.
Outputs	Desirable output: *GDP*	The Gross Domestic Product of the city, with all the values being converted into 2010 constant price.
	Undesirable output: CO_2_	Carbon emissions from energy combustion (fossil fuels, electricity, and heat) of the city.

**Table 3:** The cities with the largest or smallest carbon emissions in China.

**City**	**2005**	**2006**	**2007**	**2008**	**2009**
City with largest carbon emissions	Shanghai	Shanghai	Shanghai	Shanghai	Beijing
City with smallest carbon emissions	Longnan	Longnan	Longnan	Longnan	Longnan
**City**	**2010**	**2011**	**2012**	**2013**	**2014**
City with largest carbon emissions	Shanghai	Shanghai	Shanghai	Shanghai	Shanghai
City with smallest carbon emissions	Longnan	Longnan	Longnan	Lijiang	Lincang
**City**	**2015**	**2016**	**2017**	**2018**	**2019**
City with largest carbon emissions	Shanghai	Shanghai	Shanghai	Shanghai	Shanghai
City with smallest carbon emissions	Lincang	Lincang	Lincang	Lincang	Lincang
